# Melanoma progression is associated with NK cell exhaustion

**DOI:** 10.1186/2051-1426-2-S3-O6

**Published:** 2014-11-06

**Authors:** Ines Pires da Silva, Anne Gallois, Sonia Jimenez-Baranda, Ana Anderson, Vijay Kuchroo, Iman Osman, Nina Bhardwaj

**Affiliations:** 1 NYU Cancer Center, New York, NY, USA; 2 Icahn School of Medicine at Mt Sinai, New York, NY, USA; 3 Covance, Madrid, Spain; 4 Center for Neurologic Diseases, Brigham & Women's Hospital, Boston, MA, USA; 5 NYU Langone Medical Center, New York, NY, USA

## Introduction

The immunoregulatory protein T cell immunoglobulin- and mucin-domain-containing molecule-3 (Tim-3) contributes towards T cell exhaustion in several chronic diseases, including melanoma [1]. NK cells from the latter were shown to be functionally impaired/exhausted, as they failed to proliferate, produce cytokines or kill target cells. In addition they down regulated activating receptors (NKG2D and NKp46) and upregulated inhibitory receptors (KIRB1, KIRNKAT2 and Tim-3). Notably Tim-3 blockade reversed this exhausted phenotype, implicating this molecule as a major checkpoint inhibitor in advanced melanoma [2]. To further evaluate NK cell phenotype and function as a consequence of progressive melanoma, we monitored NK cells from a large cohort of patients with stage I-IV melanoma tested the association between NK cell phenotype and clinicopathological variables associated with melanoma prognosis. Expression of MICA (NKG2D ligand) and HMGB1 (Tim-3 ligand) in the plasma/sera of our main cohort was also monitored in an independent validation cohort.

## Methods

NK cells were purified from the peripheral blood of melanoma patients. They were evaluated for the expression of activating and inhibitory receptors. Cytotoxicity was measured by Lamp-1 expression. IFN-γ production was measured after 4h stimulation with rhIL-12. Proliferation was quantified by CFSE after 6 days in the presence of rhIL-2. MICA and HMGB1 expression on patients' plasma/sera was measured by ELISA. Event-time distributions were estimated with the use of the Kaplan-Meier method. Two tailed t-test unpaired was used to compare samples from different stages.

## Results

**Figure 1:**
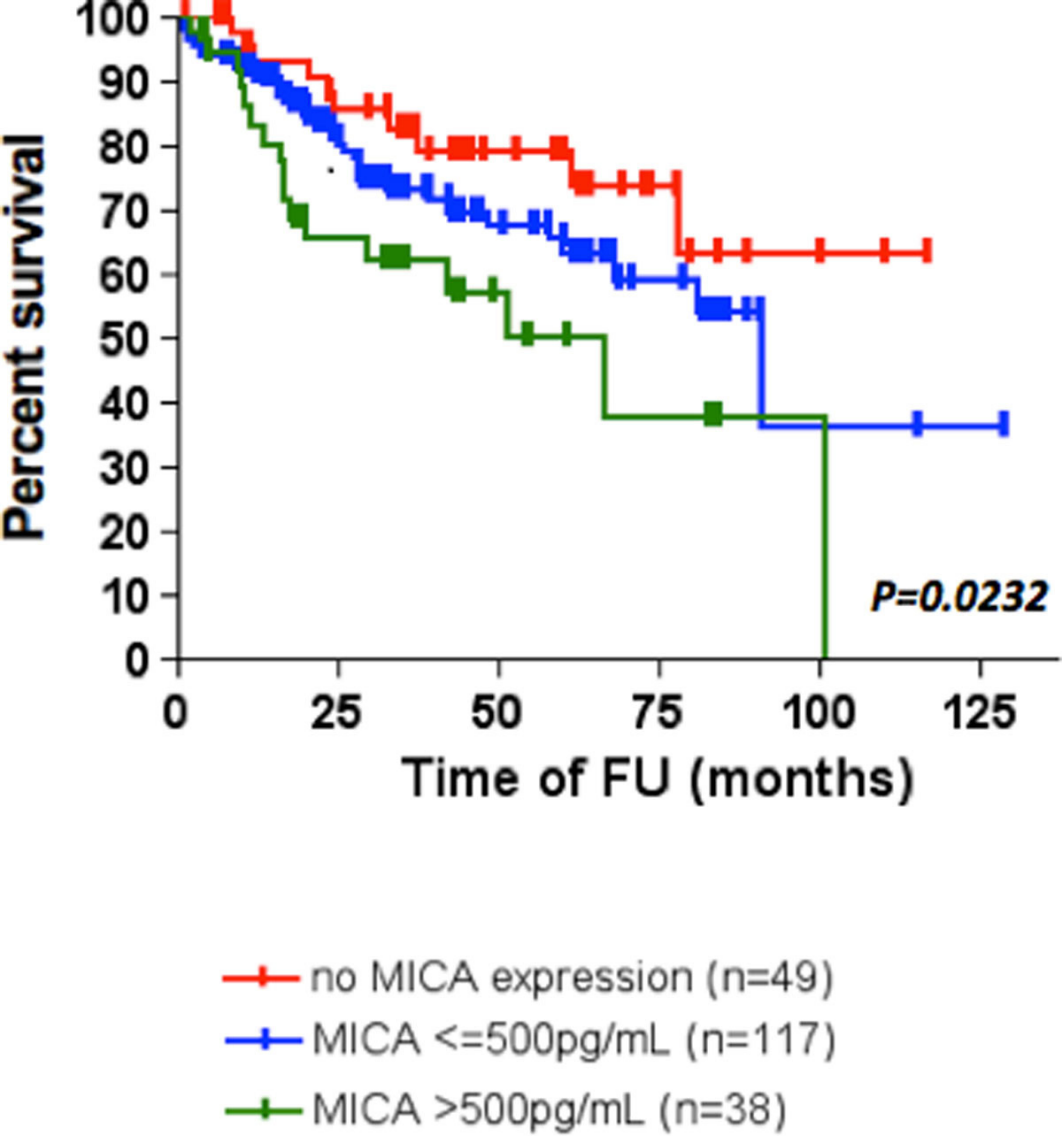
Independent validation cohort

NK cells gradually develop a phenotypic and functional profile consistent with progressive exhaustion, from stage I to stage IV characterized by: 1) up-regulation of inhibitory receptors (Tim-3, KIRB1 and KIRNKAT2); 2) down-regulation of activating receptors (NKG2D and NKp46); 3) loss of IFN-γ production, proliferation and cytotoxicity. Interestingly, the expression of Tim-3 is higher, while the cytotoxicy and IFN-ϒ production is reduced in patients with melanomas thicker than 1mm. Moreover, higher expression of Tim-3 and KIRB1, and a lower cytotoxic ability and T-bet expression is associated with local or distant metastases. Higher expression of MICA in the plasma was associated with worse prognosis, as was validated in an independent cohort (Figure 1).

## Conclusions

These data demonstrate that NK cells become progressively exhausted in the context of melanoma progression and that Tim-3 blockade possibly earlier in disease may have some benefit on innate immune function. Finally, our data suggest that soluble MICA is a potential prognostic marker which may contribute to the NK cell exhaustion through its interactions with NKG2D.

## References

[B1] Baitsch L Baumgaertner P Devevre E Raghav SK Legat A Barba L Exhaustion of tumor-specific CD8(+) T cells in metastases from melanoma patients The Journal of clinical investigation 2011 121 6 2350 60 10.1172/JCI46102 21555851 PMC3104769

[B2] Ines Pires da Silva Anne Gallois Sonja Jimenez Baranda Shaukat Khan Ana C Anderson Vijay K Kuchroo Reversal of NK cell exhaustion in advanced melanoma by Tim-3 blockade Cancer Immunol Res 2014 2 5 410 22 10.1158/2326-6066.CIR-13-0171 24795354 PMC4046278

